# The Preparation of Thin Conductive Polyimide Foils for Nuclear Targets

**DOI:** 10.1021/acsomega.4c00840

**Published:** 2024-08-05

**Authors:** Jolanta Karpinska, David Lewis, Goedele Sibbens, Yetunde Aregbe

**Affiliations:** European Commission, Joint Research Centre (JRC), Directorate G—Nuclear Safety & Security, Unit G.II.5—Nuclear Data and Measurement Standards, Retieseweg 111, Geel 2440, Belgium

## Abstract

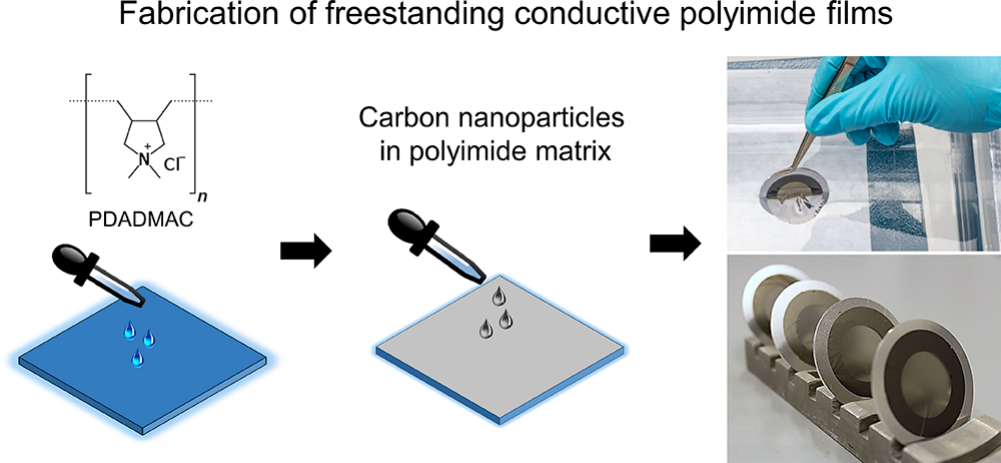

Thin and conductive plastic foils are of great interest to the target preparation and nuclear physics communities as a backing support for neutron-induced reaction measurements. This paper describes the preparation and characterization of thin, freestanding conductive polyimide films with an areal density suitable for target preparation in nuclear chemistry applications. The films were fabricated by blending a variety of graphene-based nanoparticles, a custom-made graphene suspension, and carbon nanotubes within a polymer matrix. The fabrication of freestanding polyimide films with an areal density of 30 μg/cm^2^ (∼210 nm) was both time-consuming and difficult. Here, a novel approach is described that employs a sacrificial layer and graphene material to make thin (pure and conductive) polyimide foils readily available within 24 h.

## Introduction

Thin polyimide (PI) foils are often used in nuclear chemistry experiments as backing support for the production of thin layers of radioisotopes, referred to as nuclear targets^1^ or as shielding to protect detectors from fission fragments.^2^ PI foils have excellent mechanical and chemical properties including their strength, stability, and resistance to charged particles,^1^ and they continue to be superior to other types of materials such as thin metallic foils. Commercially available PI foils are not suitable for nuclear chemistry experiments due to their relatively high thicknesses that can cause high-energy losses as particles pass through the material. PI foils with an areal density as low as 10 μg/cm^2^ can be produced via *in situ* polymerization. Due to the nonconductive nature of the PI foil, thin deposits of the element of interest are fabricated via physical vapor deposition (PVD). Although this method is known to produce thin homogeneous deposits of excellent quality, the relatively low yield and costly PVD equipment makes it prohibitively expensive.^3^ In comparison, <5% yield is expected with the PVD process, whereas >90% yield can be obtained when the targets are fabricated by molecular plating.^3^ This difference is often considered as a critical factor when working with rare and high-purity radioisotopes due to their cost.

Molecular plating is an electrodeposition method where the molecular form of an element, rather than the metallic, is deposited.^4^ Molecular plating can deliver high-quality thin layers suitable for the fabrication of nuclear targets and is therefore the currently preferred method. The backings for thin layers of radioelements that are used to make nuclear targets and to measure charged particles must be uniform, stable, and, in most cases, electrically conductive.^5,6^ At the EC-JRC Target Preparation Laboratory, aluminum foils with thicknesses of 2–20 μm, which corresponds to areal density of 542–5420 μg/cm^2^, are typically used as backing for thin deposits of actinides, such as isotopes of U, Pu, Am, and Np produced via molecular plating.^7−9^ However, due to the much lower density of PI, it is possible to fabricate freestanding foils with much lower areal density and further reduce the energy loss of particles passing through the target during neutron-induced reactions. Therefore, the necessity to produce thin and conductive foils emerged from the need to deliver thin, conductive backings of sufficient mechanical strength for high-quality deposits for use in nuclear chemistry experiments.

Significant efforts have been made to investigate different routes for the production of conductive PI films. These include metallization of PI,^10^ blending of PI with other conductive polymers,^11^ or the use of conductive fillers such as nanowires^12^ and carbon nanomaterials. Among those: carbon black, graphite granules,^13^ and carbon nanotubes (CNTs)^14^ were used to prepare conductive PI films by embedding them in a polymer matrix. Nanoparticles have been widely studied for over two decades as promising materials for transparent conductive films due to their unique properties.^15,16^ Graphene materials in particular exhibit excellent chemical stability, mechanical properties, high electrical conductivity, and unique optical behavior.^15^ These properties are of significant interest when considering suitable conductive fillers for the production of nuclear targets. The introduction of carbon nanoparticles to a polymer matrix improves their properties and should ideally have no effect on any subsequent nuclear experiments. Continuous advancement in the production of carbon nanomaterials offers an increasing range of carbon-based nanoparticles that enable or optimize a specific application. In this paper, for the purpose of producing conductive PI films, we tested a custom-made graphene dispersion with a range of carbon nanoparticles such as single layer graphene (SLG), graphene nanoplatelets (GNnP), CNTs, and superconductive carbon black (CB).

Further, the “traditional method”^17^ of producing pure and freestanding PI foils, which was first implemented in 1979,^1^ first at CBNM, then at IRMM, and today at the EC-JRC Target Preparation Laboratory,^17^ is revised. The main drawback of the traditional method was the time-consuming delamination of the foil from the substrate, as it takes several months for the foil to relax before it is ready to detach. To address this, we introduced a sacrificial layer (poly(diallyldimethylammonium chloride) PDADMAC) and adapted the existing PI production procedure accordingly. PDADMAC, a water-soluble cationic polyelectrolyte, was previously successfully employed in the production of large area (13 cm in diameter) freestanding polyvinyl formal films^18^ and was therefore a promising reagent to be used as a sacrificial layer in the immediate production of pure and conductive PI films.

## Experimental Section

### Materials

Twenty wt % in H_2_O poly(diallyldimethylammonium chloride) solution (PDADMAC) and Triton X-100 were purchased from Merck. DimethylformamideDMF), 1,2,4,5-benzenetetracarboxylicdianhydride (PMDA), and 4,4′-diaminodiphenylether (ODA) monomers were readily available. Commercially available carbon-based nanoparticles: CNTs, CB, GNnP, and SLG with specifications listed in Table 1 were purchased from VWR. E-Graphene (EGN), a homogenous suspension of graphene without additives in DMF, was acquired from Sixonia Tech. The concentration was determined gravimetrically to be ∼5 mg/mL. As graphene has the tendency to agglomerate upon storage, the dispersion was used within a week of the delivery date.

**Table 1:** Supplier Specifications of Carbon-Based Materials; Dimensions: Diameter (diam) and Length (L), Specific Surface Area (SAA), and Electrical Conductivity in Siemens per Meter (S/m)

filler type	dimension	SAA m^2^/g	S/m
CNTs	diam 10–20 nm; L 5–15 μm	a	a
CB	a	64.5	a
GNnP	diam 2–10 nm; L 5 μm	20–40	80,000
SLG	1–5 atomic layer nanosheets	650–750	500–700

a Data unavailable.

### Preparation of Glass Substrates

Scratch free 100 × 100 mm glass plates were used as substrates for thin film fabrication. Prior to thin film deposition, the glass substrates were washed with deionized water (DI), rinsed with methanol, and air-dried. Finally, the nanoETCH plasma system was used to generate oxygen plasma to remove organic contaminates and to increase the surface energy of the substrate, improving its wettability and thus coating of the glass surface.

### Preparation of Freestanding PI Films on a Fast Release Layer

To enable immediate release of the PI film from the glass plate, a 5 wt % in H_2_O PDADMAC solution was used as a sacrificial layer: 3 mL of solution was deposited on top of the cleaned glass substrate using a syringe equipped with a 0.45 μm syringe filter. The PDADMAC solution was then spin-coated at 5000 rpm for 10 s at a 500 rpm/s acceleration rate. Finally, glass substrates were dried on a preheated hot plate at 70 °C for 30 s. Equimolar quantities of ODA and PMDA monomers were used to prepare a polyamic solution (PAA) in DMF in various concentrations: 8, 10, and 12 wt %, in an argon glovebox. A dual asymmetric centrifuge Hauschild SpeedMixer was used to dissolve the monomers and form PAA. A volume of 3 mL of PAA solution was deposited on top of the PDADMAC treated substrate using a syringe equipped with a 0.45 μm syringe filter. Various rpm (1000–4000) values were used during the spin coating of PAA with the aim of producing freestanding PI films having areal densities in the range of 30–600 μg/cm^2^. The lowest areal density we could achieve using the traditional^17^ and new methods for pure PI films was 30 μg/cm^2^. In our experience, the viscosity of PAA was the limiting factor. In addition, further handling of films lower than 30 μg/cm^2^ proved to be unviable due to their high failure rate.

### Preparation of Freestanding PI Films with a Conductive Filler

Nanoparticles with a mass from 0.1 to 0.25 g of CB were used to produce PI/CB foils. For the PI/CNT foils, 0.1–0.5 g of CNTs were used. The PI/GNnP foils were fabricated with 0.1–0.22 g of GNnP, and the films of PI/SLG were prepared by dispersing SLG particles in a range of 0.015–0.05 g. Each was dispersed in 5 g of DMF per suspension. Three drops of Triton X-100 were added to each suspension to address the problematic dispersion of carbon nanoparticles. Triton X-100 is a nonionic surfactant, which was previously reported to enhance the dispersion of carbon materials for various applications, including CNTs in a polymer matrix.^19^ It was demonstrated that, in the presence of an inert gas, such as N_2_ or Ar, Triton X-100 evaporates without decomposition. However, in the presence of O_2_, the degradation products are mostly gaseous.^20^ It is expected therefore that during thermal curing of PI little to no residue of Triton X-100 remains in the thin PI films.

For optimal dispersion of particles, each suspension was mixed on a magnetic stirrer for at least 6 days. Then, in an argon glovebox, equimolar quantities of ODA and PMDA were added simultaneously to each suspension containing CB, CNT, and GNnP to reach a 12 wt % concentration of PAA and for the SLG suspension to reach concentrations of 8, 10, and 12 wt %. All suspensions were mixed further overnight in an argon glovebox.

#### PI/EGN

As water is known to be an impurity in the formation of PI, an EGN dispersion in DMF was dried using molecular sieves prior to use. Four times 5 g of EGN suspension was then decanted, and equimolar quantities of ODA and PMDA were added to reach a 6, 8, 10, and 12 wt % concentration of PAA. A dual asymmetric centrifuge Hauschild SpeedMixer was used to dissolve the monomers and form PAA in EGN dispersion.

Finally, all suspensions were spin-coated at various rotational speeds (1000–4000 rpm) on top of the PDADMAC-coated glass substrate to fabricate foils in various thicknesses.

### Thermal Curing and Film Delamination

Thermal polymerization of PAA on top of the PDADMAC layer was carried out in a Despatch LCC/LCD series oven. To investigate the stability of the sacrificial layer, three different substrates with PDADMAC and PAA layers underwent thermal treatment at 220, 240, and 260 °C, respectively. These temperatures were selected based on the previously observed thermal stability of PDADMAC.^21^ Only PI foils cured at 220 °C were easily delaminated in water, demonstrating that the higher temperatures adversely affect the stability of the sacrificial layer. Thus, 220 °C was selected.

**Figure 1:**
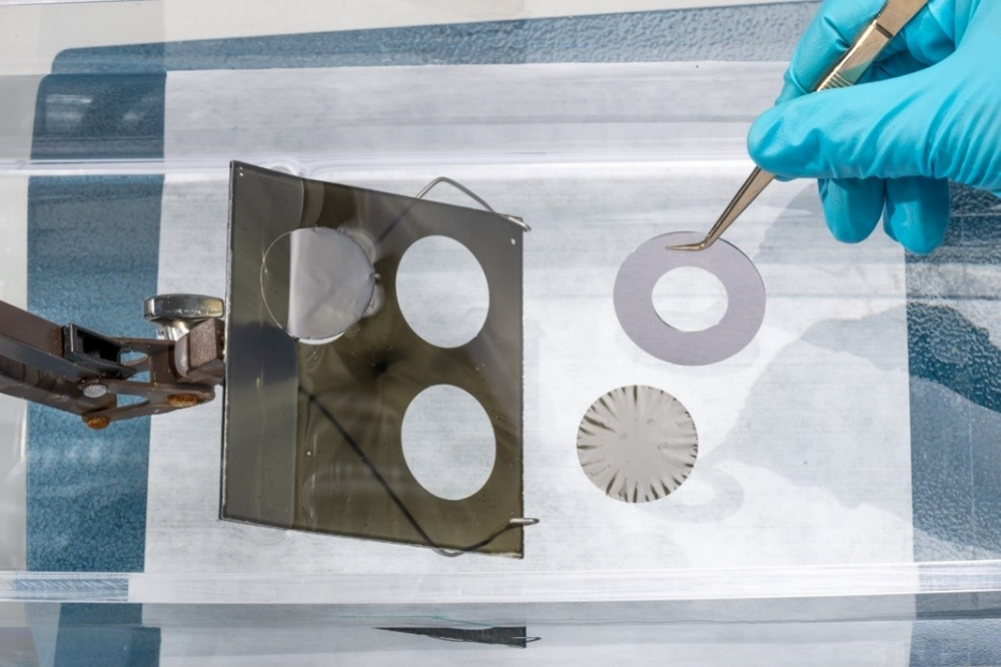
Floating of PI/SLG foils in a water bath and mounting on circular Al rings.

**Figure 2:**
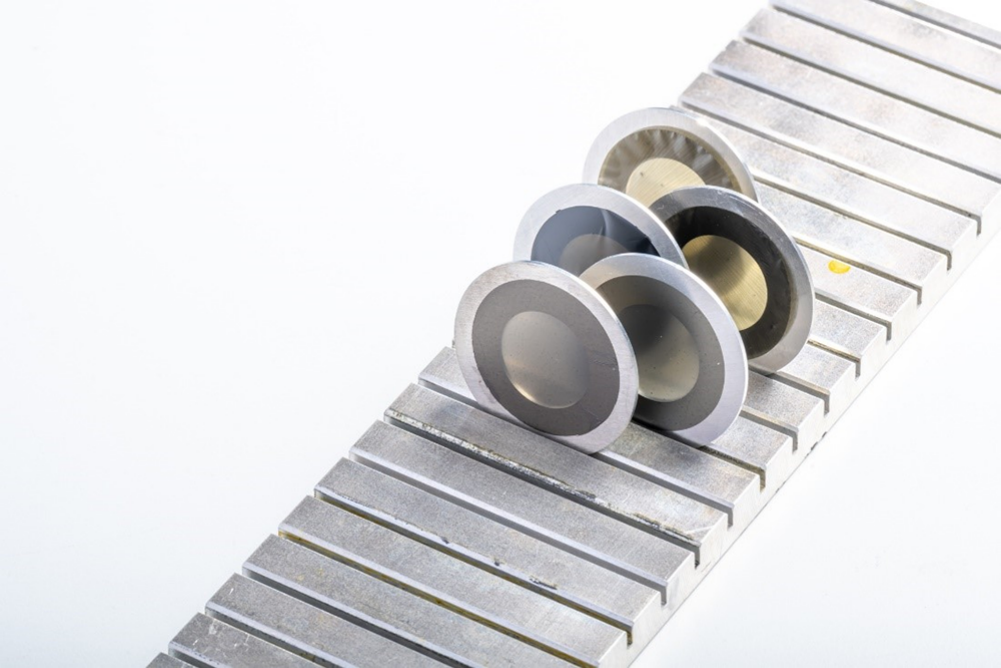
PI/SLG foils were mounted on Al rings.

It is expected that more than 90% conversion of PAA to PI is achieved above 200 °C resulting in a film that is strong enough to detach from the glass substrate.^22^ After the first thermal treatment was completed, circular films with a 30 mm diameter were cut using a circular blade, floated off the glass substrate in a water bath, Figure 1, and mounted on Al rings with a 40 mm outer and 20 mm inner diameter and a 1 mm thickness, Figure 2. In the traditional method,^17^ where no sacrificial layer was used and one thermal treatment at 350 °C was performed, the PI films were stored for a minimum of three months before it was possible to detach them in a water bath. In the new method, the use of a PDADMAC layer enables the immediate release of the film from the glass substrate after polymerization is completed. The mounted films underwent a final thermal treatment at 350 °C to complete polymerization and yield high-quality films.

### Carbon Foils (C-GSI)

Freestanding carbon foils with an areal density of 120 μg/cm^**2**^ were fabricated at the Target Laboratory of GSI Helmholtzzentrum für Schwerionenforschung GmbH in Germany^23^ and used as received.

## Characterization

### X-Ray Photoelectron Spectroscopy (XPS)

Samples were sent to the Sustainable Materials Lab at KU Leuven to be analyzed using XPS. The spectra were recorded on a Kratos Axis Supra X-ray Photoelectron Spectrometer employing a monochromated Al Kα = 1486.6 eV, 120 W) X-ray source, hybrid (magnetic/electrostatic) optics (slot aperture), hemispherical analyzer, multichannel plate, and a delay line detector (DLD) with a takeoff angle of 90°. The analyzer was operated in fixed analyzer transmission (FAT) mode with survey scans taken using a pass energy of 160 eV and a step size of 0.5 eV. All scans were acquired under charge neutralization conditions using a low energy electron gun within the field of the magnetic lens. Five areas of 300 × 700 μm per sample were analyzed. The resulting spectra were processed using CasaXPS software. The binding energy was referenced to the oxygen 1s peak maximum at 532 eV.

### Areal Density (Areal ρ)

High-precision weighing was used to calculate the areal density of pure and conductive PI foils mounted on Al rings. Weighing was performed using the substitution method (SUUS, also known as ABBA), where the mass of an unknown sample is determined through comparison with a similar mass of standards (E2, SN: G045621, 1 mg–200 g) as defined by OIML.^24,25^ The balance was used as a comparator, and thus, any drift and linearity effects during the weighing sequence do not influence the ultimate weighing result. Temperature, pressure, and relative humidity were recorded and included in calculations to correct for air buoyancy.

### Mechanical Strength

**Figure 3:**
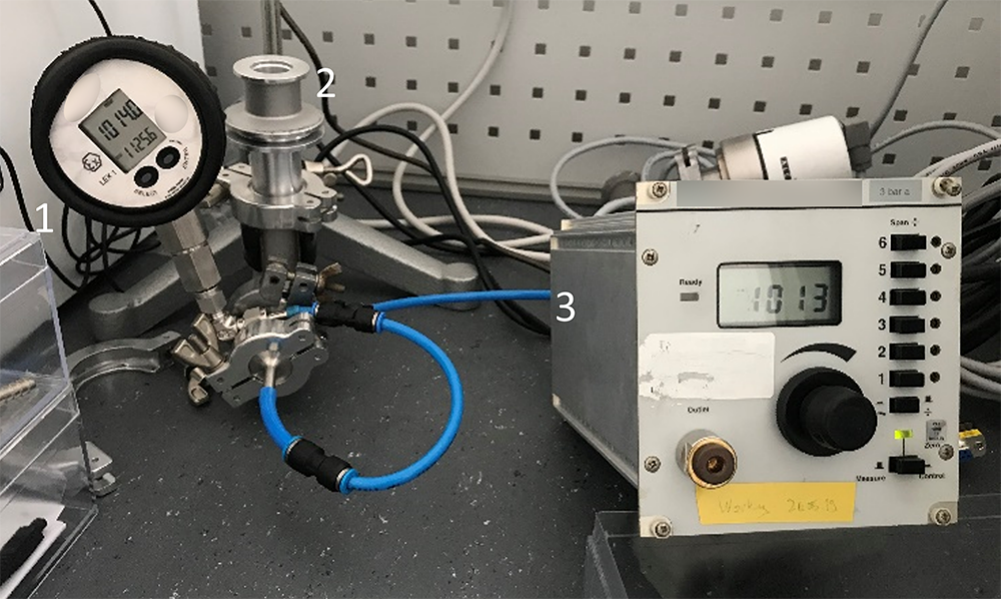
An in-house experimental setup for investigating the mechanical strength of the foils: (1) pressure indicator, (2) sample cell, and (3) pressure controller.

The mechanical strength of pure PI and PI/C foils mounted on Al frames was tested by measuring their rupture and calculating the differential pressure between atmospheric and rupture pressure. Foils with a 30 mm diameter and 30–220 μg/cm^2^ areal density were tested. Pure PI foils were used as a reference material. For this purpose, an in-house experimental setup, shown in Figure 3, was assembled. A vacuum pump was used to generate low pressure in the system, and then a DPI 530 pressure controller was used to gradually lower the pressure by 20 mbar increments until a rupture was observed.

### Sheet Resistance (Rs) and Conductivity Measurement

Thesheet resistance of PI/C foils was measured using the Ossila Four-Point Probe system directly on the glass substrates. For the conductivity measurements, freestanding films with a 30 mm diameter were used. The theoretical film thickness was calculated from the areal density of each circular film.

### Microscopy

Microscopyimages were acquired using a Leica DM 4000 M optical microscope and Park System XE-150 atomic force microscope (AFM) operated in the amplitude modulation intermittent contact mode. Image analysis was performed with the XEI (Park Systems Corp., Suwon, KR) software package.

### Alpha-Particle Transmission

The energy loss of alpha particles after passing through a PI/C foil was measured by high-resolution alpha-particle spectrometry. A 50 mm^2^passive-implanted planar silicon (PIPS) detector was used to measure the alpha particle energy spectrum of an ^241^Am source with characteristic energy peaks at 5544.5, 5485.56, and 5442.8 keV. The alpha particle energy spectrum was first measured without any foil on top of the ^241^Am source and then consecutively with each foil for 500 s. The alpha-particle spectra were analyzed in the energy region from 4000 up to 6000 keV.

## Results and Discussion

### Implementation of a Sacrificial Layer to the Production of PI Foils

To assess if the PDADMAC layer is removed by DMF during the deposition of PAA solution, DMF was spin-coated at low rotational speed on top of the sacrificial layer. When PDADMAC is spin-coated on a glass substrate at low rotational speeds, it forms a visible dendritic pattern rather than a smooth film. This property allows visual confirmation that the PDADMAC layer is not affected by DMF as the dendritic pattern can still be observed. Conversely, spin coating of water dissolves the PDADMAC layer and removes the dendritic pattern.

**Figure 4:**
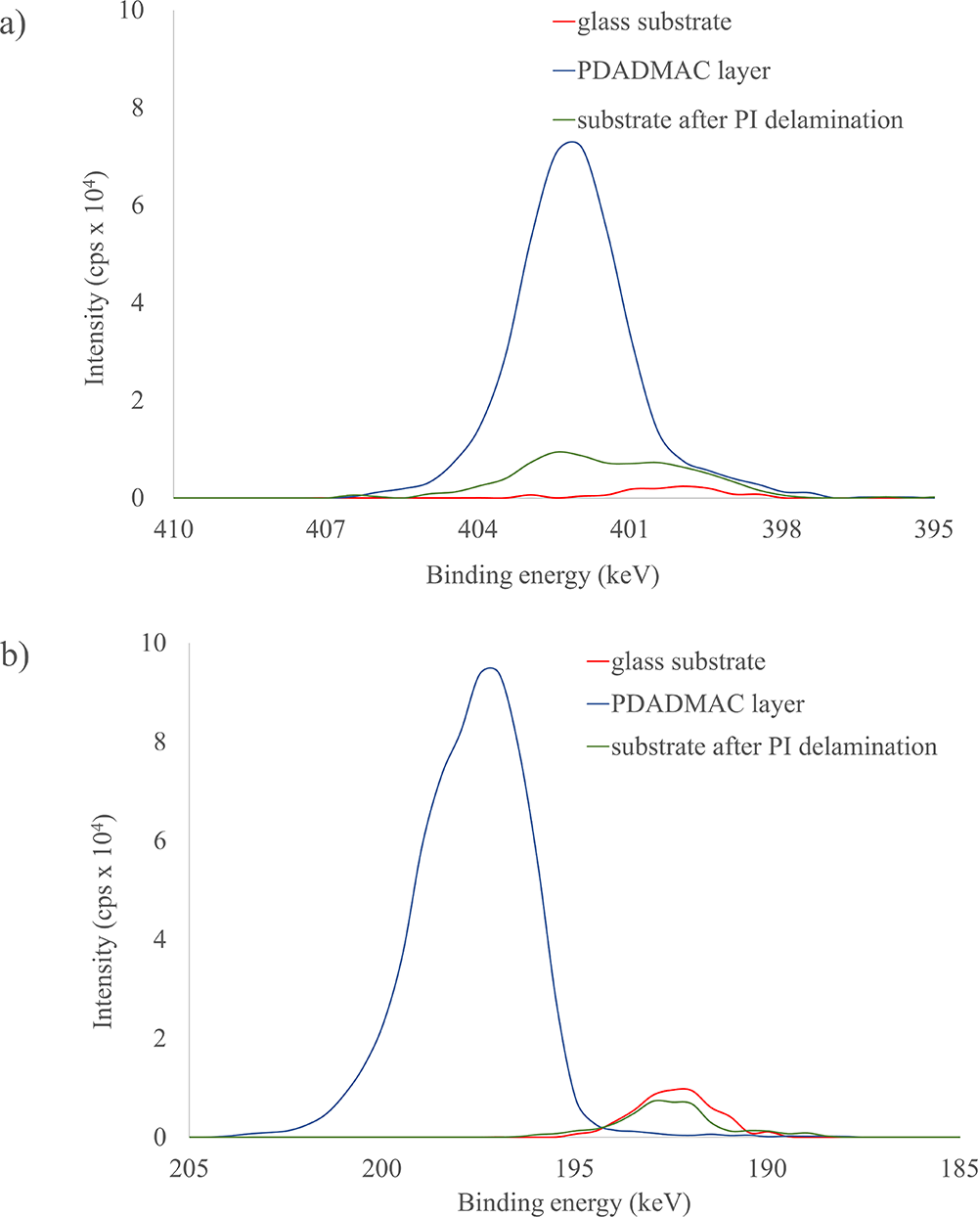
(a) N 1s spectrum (top) and (b) XPS Cl 2p spectrum (bottom) of the PDADMAC layer and glass substrates used in PI foil production.

It was previously demonstrated that a monolayer of PDADMAC bonds strongly to a modified, hydroxyl rich silicon surface and remains on the silicon wafer.^18^ Such substrates may be successfully reused in the further production of thin films without the necessity of reapplying the PDADMAC layer. In the case of PI foil fabrication, glass substrates, which first undergo cleaning with oxygen plasma, are commonly used. To investigate the removal mechanism of the PI and PI/C foils from the PDADMAC layer: (1) clean glass substrate, (2) PDADMAC coated, and (3) glass substrate after PI foil delamination in water bath were measured by XPS. The regions for the N 1s and Cl 2p peaks were chosen as characteristic for the identification of a PDADMAC polyelectrolyte.^26^ The N 1s spectrum of a PDADMAC layer shows a single nitrogen environment at 402 eV that is attributed to the quaternary ammonium functional group (Figure 4a). The same peak is not present in the spectra of a glass substrate after PI foil removal. Further, in the region for chlorine (Figure 4b), a Cl 2p enveloping peak at 198 eV is only visible for the PDADMAC-coated substrate and corresponds to the Cl 2p_3/2_ and Cl 2p_1/2_ orbital split doublet.^27^ Again, this peak is not observed for a glass substrate, which indicates that the PDADMAC layer is completely removed from the substrate in a water bath during the floating process of the PI foils.

### Production of Conductive Foils

As the conducting behavior strongly depends on the distance between the aggregates, a sufficient load and dispersion of nanoparticles are critical for the fabrication of conductive foils. All nanoparticles used were blended with 8, 10, and 12 wt % concentration of PAA. An E-Graphene dispersion was additionally used to fabricate ultrathin conductive films using 6 wt % PAA. However, only the SLG nanoparticles and E-Graphene dispersion resulted in freestanding foils in the range of concentrations used. GNnP and CNT formed freestanding foils with 12 wt % PAA having no visible agglomerates. Although multiple attempts were made to develop a reproducible method, the dispersion of both (GNnP and CNT) was problematic and often produced visibly clustered particles resulting in a heterogeneous and nonconductive layer. The dispersion of C particles is a known issue, which arises from the nonreactive nature of their surface.^28,29^ Although the addition of a surfactant, Triton X-100, to the PAA/C matrix improved particle dispersion, it did not result in the consistent production of freestanding foils. Table 2 contains areal density values calculated from substitution weighing of freestanding PI/C composites that were produced over the course of multiple experiments. Freestanding foils with a broad areal density range were prepared using either SLG nanoparticles or an EGN dispersion. Further, with the EGN dispersion, foils with similar thicknesses (60–650 μg/cm^2^) to those obtained for pure PI foils were produced. It is important to note that the use of an EGN ensures a uniform dispersion of particles, which is often difficult to achieve with SLG nanoparticles. The use of GNnP and CNTs nanoparticles resulted in freestanding films with the lowest areal density values of 868 and 424 μg/cm^2^, respectively.

**Table 2:** Areal Density Range and Roughness Parameters (Rq and Ra) of Foils Produced According to the New Method

foil type	areal ρ (μg/cm^2^)	Rq (μm)	Ra (μm)
PI/CNT	420–645	0.273	0.216
PI/GNnP	868–970		
PI/SLG	195–790	0.139–0.333	0.110–0.260
PI/EGN	70–230	0.083	0.062

### Morphology

**Figure 5:**
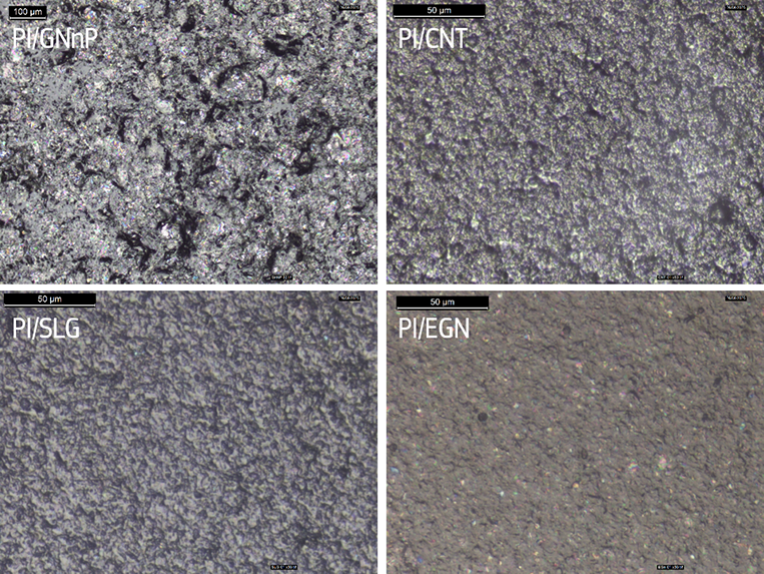
Surface morphology of the PI/C composites.

Figure 5 shows optical images of the PI/C foils prepared on glass substrates. The largest aggregates were observed on the surface of PI/GNnP foils. The images of PI/CNT and PI/SLG show similar, overlapping clusters of particles. The PI/EGN foil surface displays the most homogeneous layer where the finest dispersion of particles in a PI matrix was achieved. The roughness of the surface, which in the case of PI/C foils is determined by the lateral particle size and their dispersion, has a significant impact on the adhesion of the deposited layer.^30^ In general, a rough surface favors adhesion; however, it may also contribute to the thickness inhomogeneity of very thin deposits (<5 μg/cm^2^). Therefore, the root-mean-square roughness (Rq) and the roughness average (Ra) for selected foils were calculated, and the data are listed in Table 2. For reference and comparison, the Rq and Ra values of pure PI foils were also calculated and range between 0.0018–0.0032 and 0.0022–0.0068. As expected, the Rq and Ra values were the lowest for PI/EGN foils, indicative of a more uniform surface. The PI/CNT and PI/SLG foils show higher values, yet within a similar range. The variation in roughness parameters between specific batches for PI/SLG foils likely results from the irreproducible dispersion of particles.

### Conductivity

**Figure 6:**
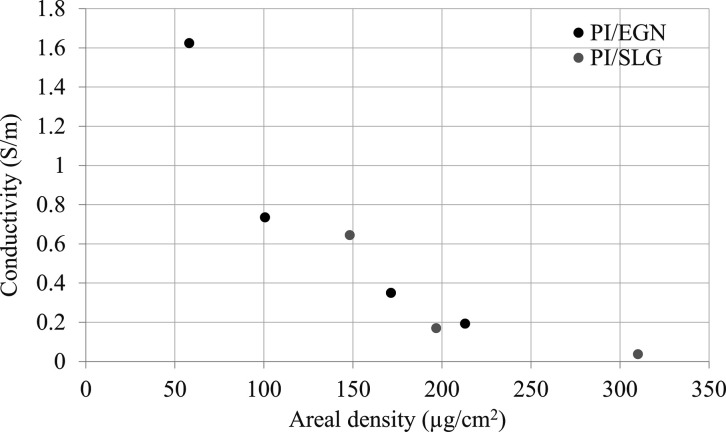
Conductivity of PI/EGN and PI/SLG foils with initial concentration of graphene particles of 5 mg/mL as a function of areal density.

The initial concentration of PAA and the spin-coating speed determines the thickness/areal density of the PI matrix, which along with the type and amount of conducting material will define the conducting behavior of the PI/C foils. Further, the optimal amount of conductive filler in the polymer matrix will determine mechanical properties such as the flexibility and strength of the foil. It was observed that, although a higher amount of particles produces a higher electrical conductivity, it also results in the formation of brittle foils that tear easily upon removal from the substrate. The conductivity values shown in Table 3 were calculated from the measured sheet resistance of individual foils. At the lowest concentration of 18.9 mg/mL for GNnP and CNT particles, the foils exhibited no conductivity. It required 42.5 mg/mL GNnP and 44.5 mg/mL CNT mixed with a polymer matrix to form a conductive layer. In comparison, conductive foils were produced from just 3 and 5 mg/mL concentrations of SLG particles and EGN dispersions. Graphene has the advantage of having a very high surface area (650–750 m^2^/g) compared to other carbon-based materials such as GNnP (20–40 m^2^/g) or MWCNT.^31^ A high surface area aids in the formation of structures with greater particle overlap, allowing electrons to pass through them, resulting in a conductive layer. In practice, this means that, for materials with a larger surface area, such as SLG, a much lower load of carbon particles is required to form a conductive foil. Specifically, when compared to SLG and EGN, 10-fold more GNnP and CNT particles are needed to yield a conductive layer. This higher load of GNnP and CNT particles resulted in an order of magnitude larger conductivity values relative to SLG and EGN, Table 3. The effect of foil thickness on the electrical conductivity is demonstrated in Figure 6. PI/EGN foils, where an ideal dispersion of nanoparticles is anticipated, show a decrease in conductivity with increasing thickness of the foil. This trend was also observed for PI/SLG foils with a similar starting concentration of nanoparticles (∼5 mg/mL). In both cases, the change in conductivity can be related to the initial concentration of the (nonconductive) PAA that determines the thickness of the film and limits charge transfer between the conductive nanoparticles.

**Table 3:** Conductivity of Selected PI/C Foils

foil type	areal ρ (μg/cm^2^)	conductivity (S/m)
PI/Au	a	2.384
C-GSI	121.43	782.9
PI/CNT	423.95	44.44
PI/GNnP	868.12	26.67
PI/SLG	148.25	0.6453
	309.95	0.5864
	590.55	0.142
	647.73	0.4066
PI/EGN	58.07	1.624
	100.56	0.7351
	171.32	0.3504
	213.01	0.1939

a The measured conductivity of a 200 nm thick Au layer on top of a PI foil.

### Mechanical Strength

**Figure 7:**
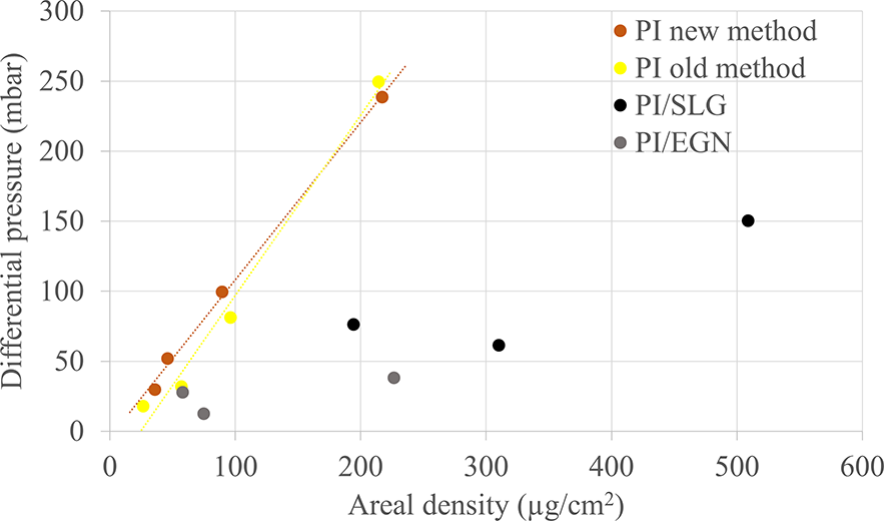
Mechanical strength of PI and PI/C foils expressed as differential pressure as a function of the areal density.

Foil strength is a critical parameter that affects handling of the films during target preparation, transport, and use. PI foils prepared by the traditional method were compared to foils fabricated by the new method to verify if the use of a sacrificial layer and modified curing step influenced foil strength. PI/SLG and PI/EGN foils were also tested. Table 4 contains the rupture pressure values recorded for the foils of interest. No significant difference was observed between the differential rupture pressure values of PI foils prepared by either method. It was therefore concluded that the new method does not negatively impact their mechanical strength, Figure 7. In the case of the thinnest foils <50 μg/cm^2^, the foils contained more pinholes when no sacrificial layer was employed. A substantial difference was noted for PI/SLG and an even greater one for PI/EGN foils. This is unsurprising, as the graphene nanoparticles are expected to impair the strength of PI/C foils by interrupting the polymer. It remains to be determined if the observed mechanical strength of the conductive foils will be sufficient for conducting molecular plating experiments. It is intended to correlate these values with the aim of introducing a pressure test as a preliminary assessment of the foil’s strength.

**Table 4:** Rupture Pressure Values Measured for PI and Conductive Foils

PI^a^		PI^b^		PI/SLG		PI/EGN	
μg/cm^2^	Δ*p* mbar	μg/cm^2^	Δp mbar	μg/cm^2^	Δp mbar	μg/cm^2^	Δp mbar
35.9	29.7	26.6	17.9	194.3	76.2	58.07	27.8
46	52	57.1	32	310	61.4	74.87	12.5
89.4	99.6	96.2	81.2	508.9	150.4	226.41	38.2
217	238.5	214.1	249.6				

a Foils prepared by the novel method on the PDADMAC layer.

b Foils prepared by the traditional method.^17^

### Alpha-Particle Transmission

**Figure 8:**
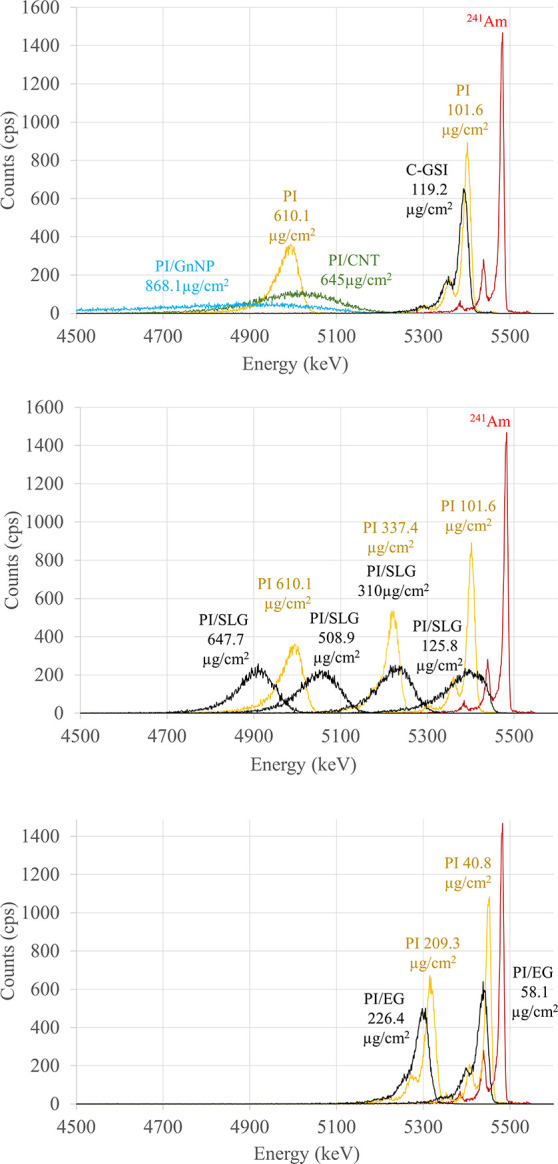
Alpha particle energy spectra of a ^241^Am source (red) covered with PI foils with different areal densities (yellow) in comparison to conductive foils.

To estimate the applicability of PI/C foils as nuclear backings, the energy loss of alpha particles emitted from a ^241^Am source was measured as they pass through each foil type with varying densities. The energy loss of each conductive foil was compared against pure carbon (C-GSI) and PI foils with similar areal densities. Figure 8 shows the alpha particle spectra of PI/C foils that were placed on top of a ^241^Am source. The ^241^Am source shows three characteristic peaks, with the most intense one at 5481 keV. A clear trend is observed for all PI and PI/C foils, where the energy loss of alpha particles increases with the thickness of the foils. By comparing the energy loss for pure PI and PI/C composites in the same areal density range, it was observed that the energy resolution, and not the peak energy shift, was affected by the distribution of C particles. Considering the fixed geometry of the measurement such a result can be attributed to a nonconstant areal density across the diameter of the film, likely due to an inhomogeneous distribution of nanoparticles. Alpha particles passing through such a film will have variable track lengths, resulting in a broader energy peak. Where characteristic peaks for the ^241^Am source can still be observed, the energy loss for a C-GSI (119.2 μg/cm^2^) foil is 87 keV and for PI foils (40.8, 101.6, and 209.3 μg/cm^2^) is 30, 81, and 165 keV, respectively. This implies that thicker foils, such as PI/GNnP and PI/CNT, are not suitable as they will most likely stop fission fragments during nuclear experiments. Surprisingly, the spectra of all PI/SLG foils, including the thinnest, with an areal density of 125.8 μg/cm^2^, show the same broad peak without the characteristic peaks of ^241^Am. The PI/EGN foils show an energy loss of 43 keV (58.1 μg/cm^2^) and 181 keV (226.4 μg/cm^2^), comparable to the pure PI foils with a similar areal density.

## Conclusions

The implementation of PDADMAC as a sacrificial layer has enabled the immediate production of freestanding PI films. Further, it has also enabled the fabrication of conductive foils, which would be difficult to achieve via the traditional method. The application of conductive plastic foils as backing for nuclear targets is limited by many factors in the fabrication process and the nature of the nuclear experiment. First, the conductivity and mechanical strength of the foils are critical to the production of a thin deposit on the foil surface via molecular plating. Second, the thickness of the foil and the type of conductive material employed are critical for the nuclear experiment. These parameters excluded most of the conductive fillers considered during this research. The lateral particle size and problematic dispersion of GNnP and CNTs resulted in foils that were too thick for the intended application as well as an inability to develop a robust process. Further, SLG nanoparticles, although initially considered a promising candidate, proved to be unsuitable for nuclear experiments as they fail to allow alpha particles to pass through when produced in the thickness range of fabricated foils. The EGN dispersion, however, has the potential to be successfully employed in the fabrication of conductive foils and as a nuclear backing. The use of EGN promotes the homogeneous dispersion of particles, which leads to improved conductivity and surface homogeneity and therefore the ability to develop a reproducible method. Whether the mechanical strength and electrical conductivity of PI/EGN foils are sufficient for thin deposits remains to be addressed by molecular plating experiments.

Further work is required to assess these properties and ultimately the applicability of PI/EGN foils as substrates in the production of thin deposits of actinides. Due to their similar chemical properties, the intention is to test first cerium oxide, followed by depleted uranium and then actinides and other radioisotopes of interest.

## Supporting Information Available

Elemental composition determined from XPS transmission corrected raw data (PDF)
