# Supplementary material for: The Preparation of Thin Conductive Polyimide Foils for Nuclear Targets
Source: ACS Omega. 2024 Aug 5;9(33):35348–55. doi: 10.1021/acsomega.4c00840 (PMC11339984; doi:10.1021/acsomega.4c00840)
Supplement: Supplementary file 1 — ao4c00840_si_001.pdf [file ao4c00840_si_001.pdf]

The preparation of thin conductive polyimide foils for nuclear targets.

Jolanta Karpinska, David Lewis, Goedele Sibbens and Yetunde Aregbe*

European Commission, Joint Research Centre (JRC), Directorate G – Nuclear Safety & Security, Unit G.II.5 – Nuclear Data and Measurement Standards, Retieseweg 111, 2440 Geel, Belgium.

*jolanta.karpinska@ec.europa.eu

Content

Table S1

Supplementary Table 1: Elemental composition determined from XPS transmission corrected raw data.

Sample id	Concentration /at. %				
	**O 1s**	**N 1s**	**C 1s**	**Cl 2p**	**Si 2p**
Clean glass substrate	50.72 ± 1.7	0.38 ± 0.17	23.39 ± 2.43	-	15.55 ± 0.55
PDADMAC coated substrate	3.77 ± 0.17	7.93 ± 0.33	78.22 ± 0.46	8.1 ± 0.25	-
Substrate after PI foil delamination	49.94 ± 1.32	0.83 ± 0.09	26.19 ± 1.63	-	16.01 ± 0.31
